# Moderating Effects of Voluntariness on the Actual Use of Electronic Health Records for Allied Health Professionals

**DOI:** 10.2196/medinform.2548

**Published:** 2015-02-10

**Authors:** Teresa ML Chiu, Benny PS Ku

**Affiliations:** ^1^Department of Rehabilitation SciencesHong Kong Polytechnic UniversityHong kongChina (Hong Kong); ^2^Hong Kong Hospital AuthorityHong KongChina (Hong Kong)

**Keywords:** health information technology, technology acceptance, user behavior, allied health, attitude towards technology

## Abstract

**Background:**

Mandatory versus voluntary requirement has moderating effect on a person’s intention to use a new information technology. Studies have shown that the use of technology in health care settings is predicted by perceived ease of use, perceived usefulness, social influence, facilitating conditions, and attitude towards computer. These factors have different effects on mandatory versus voluntary environment of use. However, the degree and direction of moderating effect of voluntariness on these factors remain inconclusive.

**Objective:**

This study aimed to examine the moderating effect of voluntariness on the actual use of an electronic health record (EHR) designed for use by allied health professionals in Hong Kong. Specifically, this study explored and compared the moderating effects of voluntariness on factors organized into technology, implementation, and individual contexts.

**Methods:**

Physiotherapists who had taken part in the implementation of a new EHR were invited to complete a survey. The survey included questions that measured the levels of voluntariness, technology acceptance and use, and attitude towards technology. Multiple logistic regressions were conducted to identify factors associated with actual use of a compulsory module and a noncompulsory module of the EHR.

**Results:**

In total, there were 93 participants in the study. All of them had access to the noncompulsory module, the e-Progress Note, to record progress notes of their patients. Out of the 93 participants, 57 (62%) were required to use a compulsory module, the e-Registration, to register patient attendance. In the low voluntariness environment, Actual Use was associated with Effort Expectancy (mean score of users 3.51, SD 0.43; mean score of non-users 3.21, SD 0.31; *P*=.03). Effort Expectancy measured the perceived ease of use and was a variable in the technology context. The variables in the implementation and individual contexts did not show a difference between the two groups. In the high voluntariness environment, the mean score of Actual Use was associated with Performance Expectancy (*P*=.03), Organization Facilitating Condition (*P*=.02), and Interest in Internet and Computer (*P*=.052) in univariate analyses. The only variable left in the logistic regression model was Organization Facilitating Conditions (mean score of users 3.82, SD 0.35; mean score of non-users 3.40, SD 0.48; *P*=.03), a variable in the implementation context. The factors affecting actual use were different in mandatory and voluntary environments, indicating a moderating effect of voluntariness.

**Conclusions:**

The results of this study have provided preliminary supports of moderating effects of voluntariness on the use of EHR by allied health professionals. Different factors were identified to be associated with actual use: (1) Ease of Use in mandatory environment, and (2) Organization Facilitating Conditions in voluntary environment. More studies are needed to examine the direction of moderating effects. The findings of this study have potential practical implications. In sum, voluntariness can be a highly relevant and important moderating factor not to be ignored in the design and evaluation of EHR.

## Introduction

### Background

This research aimed to study the role of voluntariness on the actual use of electronic health records (EHR). According to the Unified Theory of Acceptance and Use of Technology (UTAUT) model, voluntariness moderates the effect of *social influence* on intention to use [1]. Other than social influence, a meta-analysis study [2] found that voluntariness also moderates the effects of *ease of use* and *usefulness* on the intention to use a new information technology. Although these factors have provided evidence to explain in part the reasons of technology acceptance among health professionals, researchers in the health care field have argued that the technology acceptance models developed in business settings have not included factors unique to health care professionals [3,4]. A model that is adapted specifically to the health care context is needed [5].

Different technology acceptance concepts have been adapted for use in health care settings. Chau and Hu studied the doctor’s acceptance of telemedicine technology [3] and proposed a hierarchical conceptual structure of acceptance: the *individual context* in the inner core and the *technological context* in the middle layer, surrounded by the *implementation context* on the outermost layer. The concept emphasized the importance of individual context in order to predict doctor’s adoption of technology. Chau and Hu’s work was further adapted by Schaper and Pervan [4] in a study of allied health professionals’ intention to use information communication technologies (ICT) at work. Integrating the conceptual structure of UTAUT and Chau and Hu’s model, Schaper and Pervan [4] proposed a model that explains how *intention to use* is affected by three contexts of use: (1) *individual context* (computer anxiety, computer self-efficacy, computer attitude), (2) *technological context* (perceived usefulness and perceived ease of use), and (3) *implementation context* (social influence, compatibility, and organizational facilitating conditions). The model was validated using survey data of 2044 Australian occupational therapists. The study measured the self-reported use of ICT at work for the purposes of clinical, administrative, and professional development [6]. The results supported the direct effect of *effort expectancy* and *compatibility* on *behavior intention* but did not support the direct effect of *performance expectancy*, *social influence,* and *attitude* on intention. Recently, the UTAUT model was adapted by Venkatesh and colleagues to explain doctor’s adoption and use of an EHR system [7]. The results showed that age moderated between the acceptance and use of EHR *and effect expectancy*, *performance expectancy, social influence* and *facilitating conditions*. The adapted UTAUT model explained the intention to use better than the original model when applied in the health care context. Different explanatory models have found differing conceptual constructs of the use behaviors in health care context. One possible explanation of the differences could be the moderating effects of voluntariness. A literature review on the moderating effects of voluntariness on use behaviors is presented in the next section.

### Moderating Effects of Voluntariness on Actual Use in Technological, Implementation, and Individual Contexts

**Figure 1:**
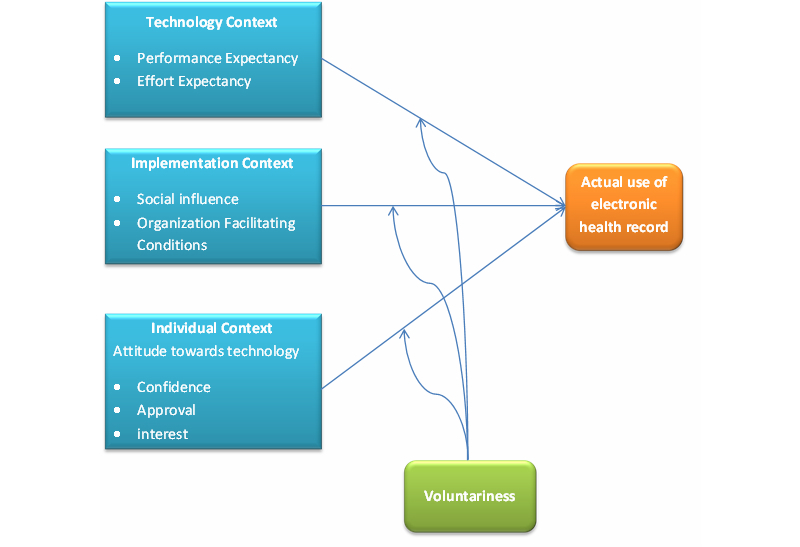
Conceptual model of the moderating effects of voluntariness on actual use.

Figure 1 represents the conceptual model of the study of the moderating effects of voluntariness on actual use. The conceptual model includes the theoretical constructs from the UTAUT [1,8], Chau and Hu’s framework [3], and Schaper and Pervan’s model [4,6]. The three contexts that influence actual use are *technological context*, *implementation context*, and *individual context*. This study focuses on *actual use* instead of *intention to use* of EHR because there is limited empirical evidence of the moderating effect of voluntariness on actual use [2]. The model also proposed the moderating effects of voluntariness in each context.

In the technology context, *perceived usefulness* and *perceived ease of use* are two main factors that influence *actual use*. According to the UTAUT, *effort expectancy,* and *performance expectancy* are the main factors that influence *behavioral intention,* which in turn predicts *actual use* of technology systems. Voluntariness does not moderate the technology context according to UTAUT [1,8]. However, in a meta-analysis of studies conducted in education and business contexts, the results showed that voluntariness moderated the effects of *ease of use* and *usefulness* on intention to use [2]. Such effects were stronger in a highly volunteer-driven environment (voluntary use) than in a low volunteer-driven environment (mandatory use). Similar findings have been reported in studies conducted in health care settings. In highly volunteer-driven environment, studies showed that *perceived ease of use* but not *perceived usefulness* significantly predicted *intention to use* a new EHR by physicians [9], and a modified case management system by health and social service professionals [10]. In both studies, the results showed that *intention to use* was predicted by *perceived ease of use* (*P*<.05) but not *perceived usefulness* (*P*=ns). However, in a study of the mandatory use of a homecare telemonitoring system in Spain, Asua and colleagues [11] found that the intention to use the system by physicians and nurses was predicted by *perceived usefulness* (*P*=.02) but not *ease of use* (*P*=ns). Contrary to these studies, Gangnon and colleagues [12] found that neither *perceived usefulness* nor *perceived ease of use* was a predictor of *intention to use* of a telemonitoring system. On the other hand, the literature is more consistent when the studies were conducted in a low volunteer-driven environment. The findings showed that *perceived usefulness* and *perceived ease of use* had similar effects on *intention to use* [13-15]. The technologies evaluated in the studies included a barcode system for bed-side medication administration [13], e-ICU technology by nurses [14], mobile health record by homecare nurses in Ontario, Canada [15], an EHR system by doctors [7], and a health information system by administration and medical staff in Greece [16]. All four studies found that *intention to use* was predicted by *perceived usefulness* (*P*<.05). Only one study evaluated the actual use of an EHR system. The results showed that perceived usefulness (*P*<.05) but not perceived ease of use (*P*<ns) predicted actual use [9]. In sum, the literature supported the moderating effects of voluntariness in technology context. Although the direction of moderating effect in highly volunteer-driven environments remains inconclusive, their effects are likely to be stronger than those of low volunteer-driven environments.

In the *implementation context*, *social influence* and *facilitating conditions* are two factors that influence *actual use*. According to the original UTAUT [1], voluntariness moderates the relationship between *social influence* and *behavioral intention*. However, when the UTAUT model was adapted to examine the use of EHR by doctors, *age* but not *voluntariness* was found to moderate the relationship between *social influence* and *behavior intention* [7]. Contrary to the UTAUT model, studies conducted in highly volunteer-driven environments have shown different relationships. Studies showed that *intention to use* was not influenced by *social influence* but *facilitating conditions* [11,12]. Ausa and colleagues [11] found that the intention to use a system by physicians and nurses was predicted by *facilitating conditions* (*P*<.001) but not *social norm* (*P*=ns). Gagnon and colleagues [12] studied the compulsory use of a telemedicine system in a clinical trial conducted in Spain and reported that the only factor remained in the final model of logistic regression was *facilitating condition* (*P*<.001), indicating the exclusion of *social influence* (*P*=ns) from the model. On the other hand, studies conducted in low volunteer-driven environments showed that *social influence* and *facilitating conditions* had similar effects on *intention behavior* [7,13,15], and *actual use* [16]. Aggelidis and colleagues [16] reported that both *social influence* (*P*<.05) and *facilitating conditions* (*P*<.05) predicted *actual use* of a health information system. Vanketash and colleagues [7] found significant effects of social influence on intention to use (*P*<.01) and facilitating conditions on actual use (*P*<.05). Holden and colleagues [13] reported a significant predictive effect of social influence on intention to use (*P*<.01). Zhang et al also [15] found an indirect effect of subjective norm on intention to use. In sum, the literature supported the moderating effects of voluntariness in implementation context. Such effects would likely be stronger in a highly volunteer-driven environment than in a low volunteer-driven environment.

The individual context refers to a person’s attitude towards technology in general. In highly volunteer-driven health care environment, individual context seemed to have limited impact on intention to use. Gagnon and colleagues [9] reported that *computer self-efficacy* (*P*=ns) did not predict *intention to use*. Schaper and Pervan [4] also found that *attitude towards computer* (*P*=ns) did not have an impact on *intention to use*. On the other hand, individual context was found to have a positive influence on intention to use in a low volunteer-driven environment. Aggelidis and Chatzoglou [16] found that *self-efficacy* (*P*=.05) and *attitude* (*P*=.05) to use computer had significant impact on the actual use of a health information system by administration and medical staff. Although there are limited studies that examine attitude towards technology in health care settings, the literature has suggested a moderating effect of voluntariness on individual context. The effect of attitude towards technology on use behavior would likely be present in a highly volunteer-driven environment but absent in a low volunteer-driven environment.

Even though the literature supported the moderating effects of voluntariness on the use of health technology, the degree and direction of its moderating effect remains inconclusive especially in the technology and individual contexts. This study aimed to address the knowledge gap and to examine its moderating effect on the actual use of an EHR for use by allied health professionals in Hong Kong. Specifically, this study explored the voluntariness and compared the mandatory and voluntary use behaviors in technology, implementation, and individual contexts (Figure 1). Table 1 presents the research hypotheses in the three contexts.

**Table 1:** Hypotheses of moderating effects of voluntariness on technology, implementation, and individual contexts.

Hypothesis			Supported literature
**Technology context**			
	In high voluntariness environment,		
		H1 - Performance expectancy is not associated with use H2 - Effort expectancy is associated with use	[9-12]
	In low voluntariness environment		
		H4 - Effort expectancy is associated with use H3 - Performance expectancy is associated with use	[7,13-16]
**Implementation context**			
	In high voluntariness environment,		
		H5 - Social influence is not associated with use H6 - Facilitating condition is associated with use	[11,12]
	In low voluntariness environment,		
		H7 - Social influence is associated with use H8 - Facilitating condition is associated with use	[7,13,15,16]
**Individual context**			
	In high voluntariness environment,		
		H9 - Attitude towards technology is not associated with actual use	[6,9]
	In low voluntariness environment		
		H10 - Attitude towards technology is associated with actual use	[16]

## Methods

### Design and Procedure

The study was conducted in the Hospital Authority, the Hong Kong Special Administration Region. The EHR system of the Hospital Authority introduced a module called Allied Health Progress Note (AHPN) that was designed for the allied health professionals including physiotherapists, occupational therapists, and other allied health professionals. Between September 2011 and January 2012, a survey was conducted to invite physiotherapists who had taken part in the implementation of the AHPN. During the study, there were 135 physiotherapists who worked in eight hospitals that participated in the trial and implementation of the AHPN, and were all invited to take part in the survey. Ethics approvals were obtained from the Hospital Authority and the Hong Kong Polytechnic University.

### Variables/Instruments

#### Level of Voluntariness

**Figure 2:**
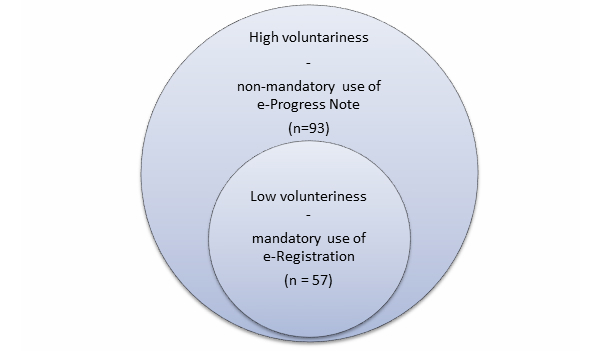
The sample distribution in the high and low voluntariness environments.

Two modules of the AHPN, *e-Registration* and *e-Progress Note*, were provided to the allied health professionals to enter health information onto the EHR system. Figure 2 presents the relationship between the two study groups in the low and high levels of voluntariness. The voluntariness scale developed by Moore and Benbasat [17] was used to define the levels of voluntariness in this study.

#### High Voluntariness Environment

The *e-Progress Note* was a nonmandatory module for all participants (n=93). Therapists had the autonomy to record the progress of their patients either using the e-Progress or paper-based health records. The nonmandatory use of the e-Progress Note module formed a high voluntariness environment in this study.

The *e-Progress Note* module was designed to support data entry in a free-text format. Designated computers with the AHPN installed were available for the therapists to type the progress notes onto the system. Multimedia Appendix 1 is a screenshot of the module.

#### Low Voluntariness Environment

The *e-Registration* was a compulsory module in in-patient units in the study sites. A subsample of physiotherapists (n=57) who worked in in-patient settings were included. The physiotherapists in the other units, such as outpatient, chose to use the e-Registration module on a voluntary basis. The mandatory use of e-Registration module by the subsample of physiotherapists formed a low voluntariness environment in this study. The e-Registration module was designed to record daily workload using electronic data entry. Instead of typing the data onto the AHPN, the therapists prepared a list of patients they have treated by affixing labels containing the hospital identity number of the patients on a paper form. The therapists then scanned the form to electronically enter the data onto the e-Registration module. Alternatively, they could delegate the scanning task to the clerical staff in the department. Multimedia Appendix 2 is a screenshot of the module.

### Actual Use of the EHR

Actual use was measured based on self-reported frequency of use. In the e-Progress Notes module, actual use was defined as the self-reported frequency of using the module in the past month. *Users* referred to the participants who selected “All of my cases”, “Most of my cases” or “About Half of my cases”. *Non-users* referred to those who selected “A few of my cases” or “None of my cases”.

In the e-Registration module, actual use was defined as the self-reported frequency of completing the scanning task in the past month. *Users* referred to participants who completed the scanning process by themselves most of the time. *Non-users* referred to those who delegated the scanning task to clerical staff.

### Technological Context

*Performance expectancy* was defined as the degree to which an individual believes that the use of the AHPN will help him/her to improve job performance [1]. In addition to the four original UTAUT items, four new items specific to the allied health workflow in the Hospital Authority in Hong Kong were added. Table 2 presents the Cronbach alpha of each measurement.

*Effort expectancy* was defined as the degree to which a person perceives the system as easy to use [1]. The four original UTAUT items were used in the study.

**Table 2:** Measurement and Cronbach alpha.

Context	Measurement	Cronbach alpha
Technology context	Performance expectancy (8 items)	.89
	Effort expectancy (4 items)	.65
Implementation context	Social influence (4 items)	.65
	Organizational Facilitating Condition (4 items)	.73
Individual context	Confidence (10 items)	.82
	Approval (10 items)	.83
	Interest (10 items)	.87

### Implementation Context

*Social influence* was defined as the degree to which an individual perceives that important others believe he/she should use an information system [1]. The original UTAUT items were used in the study.

*Organizational facilitating condition* was defined as the degree to which an individual believes an organizational and technical infrastructure exists to support the use of the system [1]. This includes factors such as management support, training, and provision of computer support. The original UTAUT items were used in the study.

### Individual Context

Individual context referred to an individual’s attitude towards the use of information technology in general. It was measured using the Technology Profile Inventory [18], which is consisted of three subscales. *Confidence* was defined as an individual’s confidence when working with computers and information systems. *Approval* was defined as the degree to which an individual feels positively about information technology as a tool to accomplish various tasks. *Interest* was defined as the intrinsic interest of an individual toward the use of information technology [19].

### Data Analysis

Univariate analysis was conducted using independent sample *t* test to compare each factor between the user and nonuser groups. Factors with *P* value less than .10 were entered to the multiple logistic regression model for analysis [20]. The SPSS (Statistical Package for the Social Sciences) software program version 19.0 was used to analyze the data.

## Results

### Participant Characteristics

A total of 93 out of 135 eligible physiotherapists participated in the study, representing a 69% response rate. Among the 93 participants, 65 of them (70%) were 31 to 50 years old and 47 (51%) were male therapists. Sixty-three (68%) had 11 to 30 years of experience, and 57 worked in in-patient settings (61%). Seventy participants reported that they spent less than 20 hours per week using personal computer (75%). Figure 2 shows the relationship of the samples in the high and low voluntariness environments. All participants (n=93) had access to the e-Progress Note module of the AHPN for use on a voluntary basis (high voluntariness environment). A subsample of the participants (n=57) who worked in in-patient units must use the e-Registration module of the AHPN (low voluntariness environment).

### Factors Associated With Actual Use in Low and Highly Volunteer-Driven Environments

Out of the 93 respondents in the high voluntariness environment 8 individuals (9%) used the e-Progress Notes. The univariate analysis results (Table 3) showed that *actual use* was associated with three factors: *performance expectancy* (*P*=.03)*, organization facilitating condition* (*P*=.02)*,* and *interest in Internet and computer* (*P*=.05). There were no association between *actual use* and the following variables: *effort expectancy, social influence,* and *the confidence, interest,* and *approval* of information technologies*.* The multiple logistic regression analysis showed that *organization facilitating conditions* (*P*=.02) was the only factor left in the final model and explained 16.5% of variance.

**Table 3:** Factors associated with actual use in high and low voluntariness environments (n=93).

		High voluntariness environment – e-Progress Note (n=93)			Low voluntariness environment – e-Registration (n=57)		
Variables		Users (N=8) Mean (SD)	Non-users (N=85) Mean (SD)	*P* value (*t* test)	Users (N=44) Mean (SD)	Non-users (N=13) Mean (SD)	*P* value (*t* test)
**Technological context**							
	Performance expectancy	3.72 (.19)	3.21 (.64)	.03^a^	3.29 (.60)	3.15 (.48)	.47
	Effort expectancy	3.68 (.52)	3.44 (.41)	.13	3.51 (.43)	3.21 (.31)	.03 ^a^
**Implementation context**							
	Social influence	3.53 (.51)	3.38 (.55)	.45	3.44 (.61)	3.21 (.44)	.22
	Facilitating condition	3.82 (.35)	3.40 (.48)	.02 ^a^	3.44 (.53)	3.35 (.41)	.59
**Individual context**							
	Confidence	3.38 (.29)	3.40 (.49)	.93	3.38 (.53)	3.24 (.39)	.38
	Interest	3.60 (.26)	3.24 (.50)	.05 ^a^	3.27 (.46)	3.11 (.48)	.28
	Approval	4.05 (.50)	3.89 (.51)	.39	3.91 (.52)	3.66 (.44)	.12
	TPI scale	3.68 (.26)	3.51 (.41)	.26	3.52 (.41)	3.33 (.34)	.15

^a^ *P*<.1 in univariate analysis; A higher score indicates a more positive rating

In the low voluntariness environment, 44 out of 57 participants (77%) completed the scanning task of the e-Registration module. The remaining participants (n=13) completed the scanning task with the help of a clerical staff. Table 3 shows the univariate analysis results. *Actual use* was associated with *effort expectancy* (*P=*.03) but not the following variables: *performance expectancy, social influence, organization facilitation condition,* and the *confidence, interest,* and *approval* of information technologies*.* A logistic regression of Effort Expectancy on Actual Use explained 34.6% of variance.

### Moderating Effects of Voluntariness on the Associations Between Actual Use and Technology, Implementation, and Individual Contexts

The study proposed a stronger moderating effect in a high voluntariness environment than in a low voluntariness environment within technology context. The results showed that the two hypotheses related to high voluntariness environment (e-Progress Note) were not supported. H1 hypothesized that performance expectancy would not be associated with use but the results showed an association between them (*P*=.03). Users of the e-Progress Note perceived the module as more useful than nonusers. H2 hypothesized an association of effort expectancy with use but the results showed no association (*P*=ns). Another two hypotheses were tested in the low voluntariness environment (e-Registration). H3 hypothesized an association between effort expectancy and use and the results supported the hypothesis and found a positive association (*P*=.03). Users of e-Registration perceived the module as more easy to use than nonusers. However, H4 hypothesized an association between performance expectancy and use behavior but the results showed no association (*P*=ns). Only one out of four hypotheses was supported. Although not all hypotheses were supported, voluntariness showed a moderating effect opposite to the proposed direction.

Another four hypotheses were proposed to test the moderating effects of voluntariness in implementation context. The study expected that the effects would be stronger in a high voluntariness environment than in a low voluntariness environment. The results supported H5 and H6 that were tested in the high voluntariness environment (e-Progress Note). H5 hypothesized no association between social influence and use, and the results showed no association as hypothesized (*P*=ns). H6 hypothesized an association between facilitating condition and use. The results supported the hypothesis and found an association as hypothesized (*P*=.02), indicating that users perceived more facilitating conditions than non-users for the use of the e-Progress Note. However, the two hypotheses tested in the low voluntariness environment (e-Registration) were not supported. H7 hypothesized that social influence was associated with use, but the results showed no association (*P*=.22). H8 hypothesized that facilitating condition was associated with use, but the results showed no association (*P*=.59). In sum, two out of four hypotheses were supported by the findings. Although the direction of association in the low voluntariness environment (H7 & H8) was opposite to the hypothesized direction, voluntariness moderates the implementation context on use behavior. The moderating effect was present but in an opposite direction as proposed.

The last two hypotheses were tested in the individual context. H9 hypothesized that in high voluntariness environment (e-Progress Note) attitude towards technology would not be associated with actual use. The results showed that there was no association (*P*=ns) in the total TPI scale but an association in the Interest subscale (*P*=.05). Users of the e-Progress Note had a greater interest in information technologies in general than non-users. H6 hypothesized that in low voluntariness environment (e-Registration) attitude towards technology would be associated with actual use. The results found no association (*P*=ns). Although both hypotheses were not supported, the impact of attitude on use behavior was likely to be present in voluntary environment and absent in mandatory environment. The results supported the presence of moderating effect but in an opposite direction.

## Discussion

The study had several limitations. The sample size was small, especially the subsample of the low voluntariness environment (e-Progress Note). If a larger sample was available, some factors might show an association with actual use such as the attitude towards technology (*P*=.15) and the approval subscale (*P*=.12). The small sample size could not provide sufficient power to confirm the moderating effects of voluntariness on the factors being tested. Furthermore, the sample was recruited from hospitals that were early adopters of the APHN. The use behavior and the moderating effect of voluntariness of early adopters may be different from that of early majority, late majority, or laggards [21]. Another limitation was that only physiotherapists were involved. The study did not include other allied health professionals such as occupational therapists, speech therapists, etc. The generalizability of the findings to other allied health professionals remains unclear. These limitations could be addressed if further studies include a representative sample of other allied health professions from more hospitals undergoing full implementation.

Despite the limitations, the study has provided some preliminary evidence that voluntariness moderates the factors affecting the actual use of APHN. Specifically, such effects were present in all three contexts but showing a moderating effect opposite to the proposed direction. The findings were most consistent with the studies in high voluntariness environment within the implementation context. The studies conducted in mandatory environment showed an association between intention to use and social influence but not facilitating conditions [11,12]. Several findings were unexpected because the moderating effect was found to be presented in an opposite direction. For example, in the voluntary use of e-Progress Note, users perceived it as more useful than nonusers, and the perceived ease of use ratings were similar in both groups. However, the literature showed that perceived ease of use but not perceived usefulness predicted intention to use in high voluntariness context [9,10]. A possible explanation of the opposite direction is that the literature measured intention to use and not actual use. The perceived ease of use may attract an initial intention to use. But after gaining some experience of using a system, the continuation of using an EHR system is motivated by the usefulness of the system instead of ease of use. Most studies reported the testing of the technology acceptance in either mandatory or voluntary situations, and few include both levels of voluntariness in the same study. Future studies of the moderating effect of voluntariness can involve the same user group that use health technology in both high and low voluntariness environments like this study.

In conclusion, the study attempted to answer the question of whether the factors predicting use behavior would differ in voluntary versus mandatory use of an EHR designed for use by allied health professionals in Hong Kong. The results have provided preliminary support of moderating effects of voluntariness on use behavior in the technology, implementation and individual contexts. Two unique factors have been identified to be associated with actual use but in different voluntariness contexts: (1) *ease of use* (technological context) in mandatory environment (low voluntariness) and (2) *organization facilitating conditions* (implementation context) in voluntary environment (high voluntariness). Interestingly, the direction of moderating effects was opposite to that reported in the literature. The literature suggested that in a mandatory environment, perceived usefulness has a greater impact on intention to use than ease of use. Further studies are needed to examine the direction of moderating effects in each context. The findings of this study have potential practical implications. A strategy that works in a mandatory environment may not work in a voluntary environment. Different strategies might be needed to promote use behavior in high and low levels of voluntariness environments. Voluntariness can be a highly relevant and important moderating factor that requires more attention in the design and evaluation of the EHR.

## Multimedia Appendix 1

Screenshot of the e-Registration of the electronic health record (with fictitious names).

## Multimedia Appendix 2

Screenshot of the e-Progress of the electronic health record (with fictitious names).

## Abbreviations

AHPN: Allied Health Progress Note
EHR: electronic Health Record
UTAUT: Unified Theory of Acceptance and Use of Technology
