# Supplementary figures and images for: Moderating Effects of Voluntariness on the Actual Use of Electronic Health Records for Allied Health Professionals
Source: JMIR Med Inform. 2015 Feb 10;3(1):e7. doi: 10.2196/medinform.2548 (PMC4376180; doi:10.2196/medinform.2548)

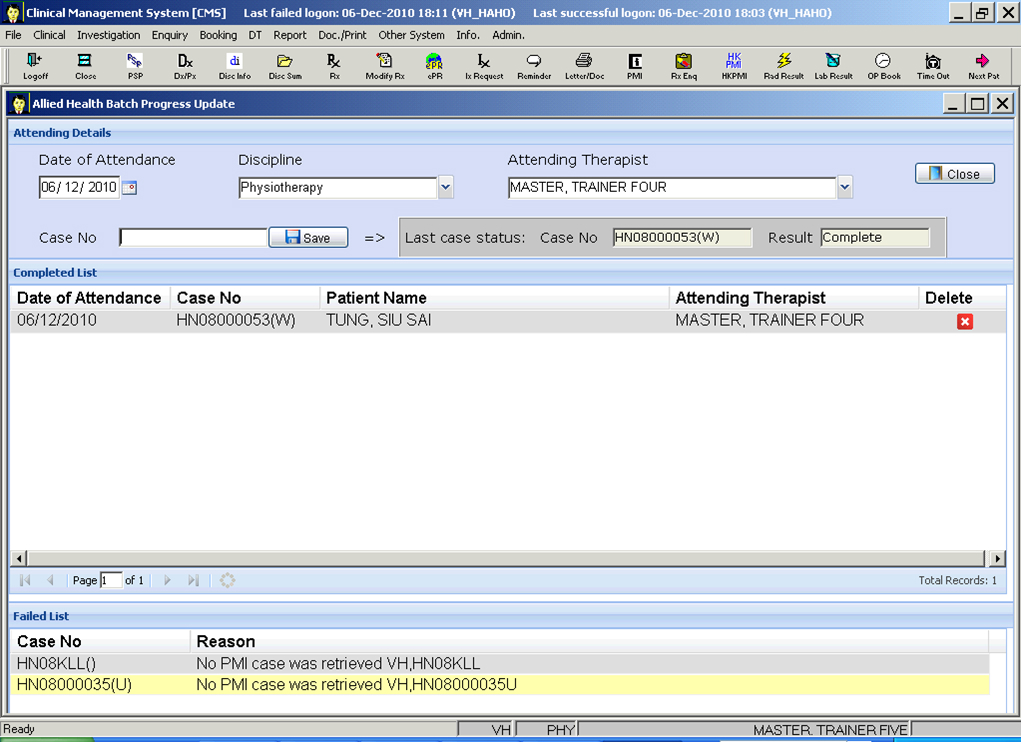

Supplement: Supplementary file 1 [file medinform_v3i1e7_app1.jpg]

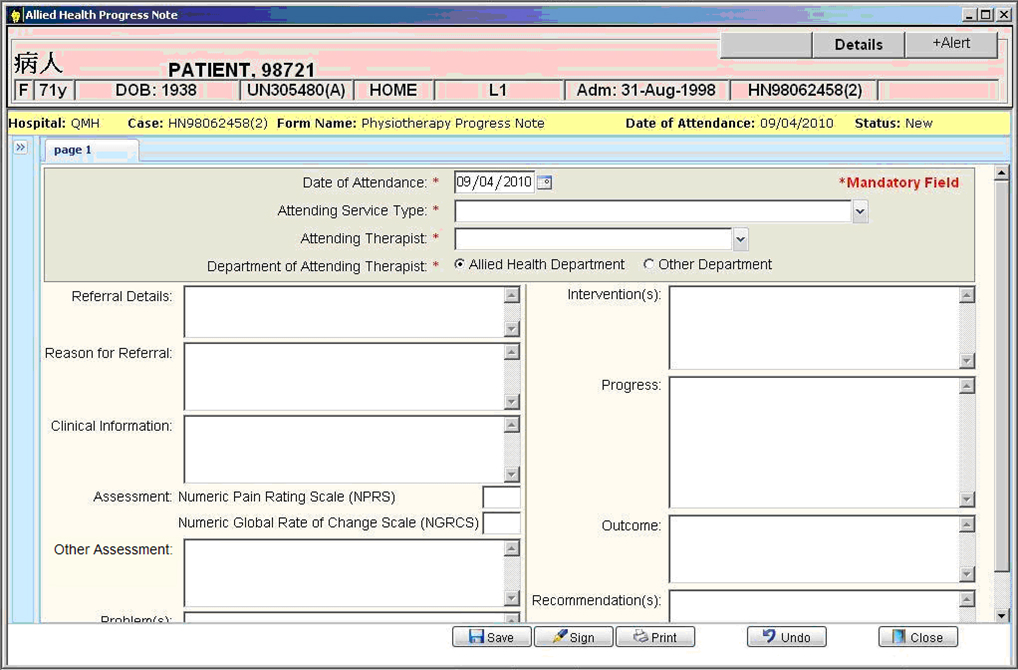

Supplement: Supplementary file 2 [file medinform_v3i1e7_app2.jpg]
